# Cohort of Patients Referred for Brugada Syndrome Investigation in an Electrophysiology Service - 19-Year Registry

**DOI:** 10.5935/abc.20180094

**Published:** 2018-07

**Authors:** Stefan Warpechowski Neto, Tiago Luiz Luz Leiria, Laura Lessa Gaudie Ley, Antonio Lessa Gaudie Ley, Luiza Zwan Dutra, Leonardo Martins Pires, Marcelo Lapa Kruse, Gustavo Glotz de Lima

**Affiliations:** Instituto de Cardiologia - Fundação Universitária de Cardiologia (IC/FUC), Porto Alegre, RS - Brazil

**Keywords:** Brugada Syndrome, Ventricular Tachycardia, Sudden Death

## Abstract

**Background:**

Brugada syndrome (SBr) is an arrhythmic condition characterized by ST-T segment abnormalities in the right precordial leads associated with a high risk of ventricular arrhythmias and sudden death. Local data regarding the clinical characteristics of patients with a typical electrocardiographic (ECG) pattern undergoing electrophysiological study are scarce.

**Objective:**

To evaluate patients with an ECG pattern suggestive of SBr referred for electrophysiological evaluation in a specialized center.

**Methods:**

Cohort study of patients referred for electrophysiological study because of an ECG pattern compatible with SBr between January 1998 and March 2017.

**Results:**

Of the 5506 procedures, 35 (0.64%) were for SBr investigation, 25 of which (71.42%) were performed in men. The mean age was 43.89 ± 13.1 years. The ECG patterns were as follows: type I, 22 (62.85%); type II, 12 (34.30%); and type III, 1 (2.85%). Twenty-three patients (65.7%) were asymptomatic, 6 (17.14%) had palpitations, 5 (14.3%) had syncope, and 3 (8.6%) had a family history of sudden death. Electrophysiological study induced ventricular tachyarrhythmias in 16 cases (45.7%), the mean ventricular refractory period being 228 ± 36 ms. Ajmaline / procainamide was used in 11 cases (31.4%), changing the ECG pattern to type I in 7 (63.6%). Sixteen cases (45.7%) received an implantable cardioverter defibrillator (ICD). In a mean 5-year follow-up, 1 of the 16 patients (6.25%) with ICD had appropriate therapy for ventricular fibrillation. There was no death. Other arrhythmias occurred in 4 (11.4%) cases.

**Conclusions:**

Most patients are men, and a type I ECG pattern is the main indication for electrophysiological study. Class IA drugs have a high ECG conversion rate. The ICD event rate was 6%.

## Introduction

Brugada syndrome (BrS) is a genetic arrhythmogenic disorder characterized by typical electrocardiographic changes of the ST-T segment in the right precordial leads (V1-V3), associated with an increased risk for sudden death due to ventricular arrhythmias, mainly polymorphic ventricular tachycardia, in the absence of structural heart disease.^1^

The BrS was first described in 1992, relates to the loss of function in the sodium ion channels of ventricular cardiomyocytes and results from the decrease in that channel amount and failure of expression, its voltage change, time-dependent action and accelerated or prolonged inactivation recovery,^2^ leading to a reduction in the sodium ion inflow and in the physiological duration of the action potential. Despite its autosomal dominant inheritance, BrS is currently known to be sporadic in two-thirds of its cases (65%),^3^ due to mutations leading to the failure of the SCN5A gene function that encodes sodium channels - initially re-written in 1998^4^ - or to other 350 pathogenic mutations in several sodium, potassium or calcium channel genes, currently representing percentages of genetic changes lower than 35%.

Because of its multifactorial etiology that involves the contribution of genetic, environmental and hormonal factors, the clinical manifestation varies, affecting mainly men (proportion of 8-9:1),^5^ with clinical onset, on average, at the age of 40 years, and major outcome of sudden death triggered by sleep, vagotonia or fever. Brugada syndrome accounts for 20% of the sudden cardiac deaths with structurally normal hearts^6^ and 4-12% of all sudden cardiac deaths.^7^

This study describes a cohort of patients referred for electrophysiological study at the Instituto de Cardiologia/Fundação Universitária de Cardiologia do Rio Grande do Sul (ICFUC), over the past 19 years (1998-2017), after finding an electrocardiographic pattern suggestive of BrS in different situations of medical care.

## Methods

This is a cohort study of patients referred for electrophysiological study at the ICFUC electrophysiology laboratory between January 1998 and March 2017. Of the 5506 studies performed in that period, 35 (0.67%) corresponded to assessment of patients with electrocardiographic pattern compatible with BrS (Brugada pattern), who were followed up from that study on.

The inclusion criteria were: absence of structural heart disease, absence of personal history of aborted sudden death, electrocardiogram (ECG) compatible with type I, II or III Brugada pattern, and electrophysiological study under a preestablished protocol of ventricular stimulation with three baseline cycles (600, 500 and 400 ms) and introduction of up to three extra stimuli. Diagnostic challenge with infusion of class IA antiarrhythmic drugs according to the Vaughan Williams classification (ajmaline at the dose of 1 mg/kg for 10 minutes or procainamide 10 mg/kg for 10 minutes) was performed in type II electrocardiographic presentations, in accordance with the most used drugs in European and American studies.^8^

From the electrophysiological study on, the patients were followed up through medical appointments at regular six-month intervals, medical record review and/or telephone contact.

### Statistical analysis

Our data bank was stored in Microsoft Excel sheets and analyzed by use of the Statistical Package for Social Sciences (SPSS) software, version 20.0 (Armonk, NY, USA: IBM Corp). The continuous variables were expressed as mean (± standard deviation) and compared by use of independent samples *t* test. The continuous variables of non-gaussian distribution were expressed as median [interquartile range (IQR)] and compared by using Mann-Whitney U test. The categorical variables were expressed as percentages and compared by use of chi-square test. The comparisons between groups were performed by using z test, with post-hoc Bonferroni analysis to identify the statistical difference. Kaplan-Meyer event-free survival analysis was performed, with percentage survival and standard error. Differences between the frequency of events over time according to the variables identified were compared by use of log-rank test. A p value < 0.05 was considered statistically significant.

### Follow-up outcomes

By use of electronic medical record review or telephone call, the occurrence of the following events was investigated: death, syncope, hospitalization due to arrhythmia, and recurrent palpitations requiring medical care. In patients receiving an implantable cardioverter defibrillator (ICD), the occurrence of shock was investigated, and, when present, the appropriateness (shock due to ventricular arrhythmia) or inappropriateness (shock due to supraventricular tachycardia, increased T-wave sensitivity or electromagnetic interference) of the event was assessed.

## Results

Of the 35 patients included in the cohort, 22 (62.85%) showed a type I electrocardiographic pattern, 12 (34.30%) showed a type II, and 1 patient (2.85%), a type III pattern. Regarding sex, 25 patients (71.42%) were of the male sex. The mean age was 43.89 ± 13.1 years, and most patients (65.71%) were asymptomatic at the time of inclusion. Regarding the symptoms, 6 patients (17.14%) had palpitations, 5 (14.28%) reported syncope, and 3 (8.57%) reported sudden death of a first-degree relative. Sixteen patients (45.7%) had induced ventricular tachyarrhythmias on stimulation - mean refractory ventricular period of 228 ± 36 ms. Eleven patients (31.4%) with type II ECG pattern received ajmaline or procainamide, and 7 of them (63.6%) changed to type I ECG pattern. Table 1 summarizes the clinical, electrocardiographic and electrophysiologic characteristics of the patients included in this study. No difference was observed between the groups with and without induced arrhythmia (Table 2).

**Table 1:** Clinical, electrocardiographic and electrophysiological study characteristics

Clinical presentations	N = 35
Men	25 (71.42%)
Age	43.89 ± 13.1 years
Asymptomatic	23 (65.7%)
Syncope	5 (14.3%)
Palpitation	6 (17.14%)
**Electrocardiographic presentations**	
Type I	22 (62.85%)
Type II	12 (34.30%)
Type III	1 (2.85%)
**Electrophysiological study**	
Ventricular tachyarrhythmia	16 (45.7%)
Refractory period	228 ± 36 ms
HV interval	49 ± 8.6 ms
Ajmaline / Procainamide	11 (31.4%)

**Table 2:** Characteristics regarding arrhythmia induction during the electrophysiological study

	With induced arrhythmia	Without induced arrhythmia	p
Number (%)	16 (45.7)	19 (54,3)	
Age	44.625 (±13.69)	43,26 (± 13,30)	0.768^¶^
Male sex	12 (75)	13 (68,42)	0.7304^¶^
**Electrocardiographic pattern**			
Type I	10	11	0.8034^†^
Type II	6	7	
Type III	-	1	
**Clinical manifestation**			
Asymptomatic	10	13	0.99^†^
Palpitations	2*	4**	0.82^†^
Syncope	3	2**	0.83^†^
FH of sudden death	2*	1	0.87^†^

* In the group of patients with induced arrhythmia, one had palpitations and sudden death in the family.

** In the group of patients without induced arrhythmia, one had palpitations and syncope. FH: family history.

¶ Student t test;

† Chi-square / Fisher exact test.

**Figure 1:**
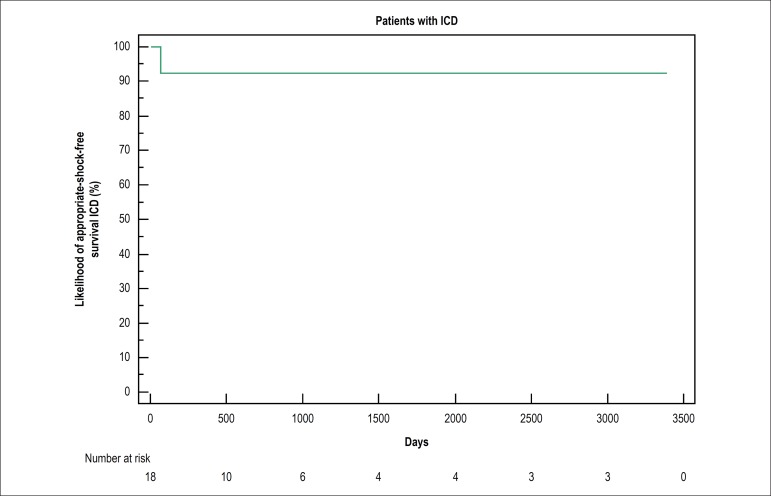
Event-free survival curve of the patients with implantable cardioverter defibrillator (ICD).

**Figure 2:**
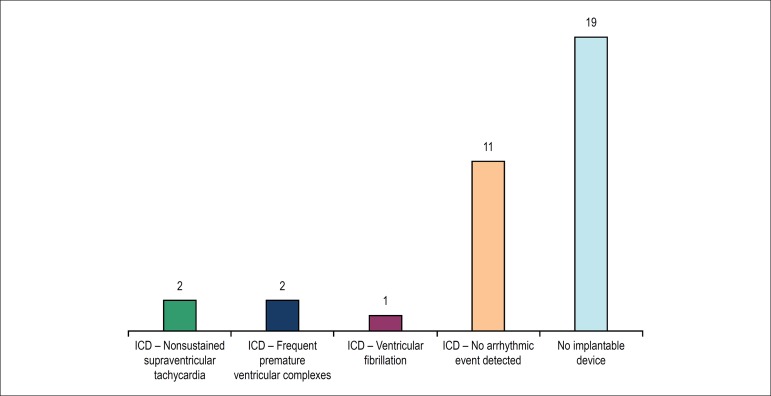
Discrimination of events in patients with implantable cardioverter defibrillator (ICD).

Sixteen patients (45.7%) received an ICD. Of those patients, only 2 had no arrhythmia triggered (reason for implantation: history of sudden death and syncope). Two patients with ventricular arrhythmia (1 with nonsustained ventricular tachycardia and another with ventricular fibrillation) refused to receive the ICD despite the clinical indication. In a mean follow-up of 5 years, 1 of the 16 patients (6.25%) who received the ICD had appropriate therapy for ventricular fibrillation, and 1 (6.25%) attended no consultation after implantation (Figure 1). No death was reported during follow-up. Four patients (11.4%) had other arrhythmic events, such as episodes of nonsustained supraventricular tachyarrhythmias and frequent premature ventricular complexes. Figure 2 shows the discrimination of events in patients with ICD.

## Discussion

The long-term event rate of patients diagnosed with BrS or electrocardiographic pattern of BrS is little known, because of the relative short time since that syndrome initial description in 1992,^1^ in addition to the limited follow-up duration of current studies, most of which no longer than 3 years.

The worldwide prevalence of BrS is heterogeneous, because of its nonpermanent electrocardiographic tracings, disparate genetic changes or undiagnosed patients. In addition, potential arrhythmic events and sudden death can occur, making long-term follow-up important to understand the disease and elaborate tools for risk stratification and therapy, mainly because of the involvement of young individuals and the long exposure to possible outcomes.

The male predominance found in this study, already reported in the initial description of the disorder as 75%,^1^ is in accordance with data from the global literature, whose percentages range according to the geographic location: 84.3% in a large Japanese cohort,^5^ 70% in a Spanish cohort,^9^ and 57.9% in a Belgian study.^10^ The proportion is maintained in geographically close populations, such as an Argentinian cohort of similar size to ours (43 patients), whose male percentage reached 85%.^11^ Likewise, the mean age of 43.89 years coincides with the findings of several populations studied, even those with larger samples,^12-14^ clearly and repeatedly showing the impairment of young individuals with high productive capacity, emphasizing the importance of the correct identification of those at higher risk based on a common epidemiological profile.

Although the history of ventricular arrhythmias of the fibrillation or tachycardia type is a predictor of mortality in patients with BrS, and the arrhythmia recurrence rates are around 7.7% per year,^14^ most of our patients were asymptomatic at the time of the electrophysiological study. If, on the one hand, asymptomatic patients without additional risk factors are currently classified as of low risk,^14,15^ on the other it is difficult to predict the potential risk based solely upon the ECG assessment, requiring a multifactorial approach in the search for other complications, such as family history of sudden death, personal history of syncope or induced arrhythmia, because the electrocardiographic pattern in isolation seems insufficient to define high risk for events.^16^

The incorporation of the advances in cardiology in the search for risk predictors has diverging results in a scenario where the identification of susceptibility is the key point, and, because therapy showed no significant changes in past years, it remains without any effective pharmacological alternative, being limited to implantable antiarrhythmic devices. Such devices are known to have a significant, although indirect, contribution to the patients’ quality of life because of their daily social or professional repercussions,^17^ adding arguments to the already challenging process of identifying its real beneficiaries.

In 2003, the assessment of 547 patients with the BrS pattern and no previous history of sudden death, with a mean 24-month follow-up, a positive electrophysiological study was associated with arrhythmic outcomes on a multivariate analysis, with a 6-fold higher risk in 2 years *versus* a 2.5-fold for the second better predictor, the previous history of syncope.^13^ In a cohort^14^ of 1029 patients (72% of men, mean age of 45 years, and 64% asymptomatic - a population profile similar to ours), the electrophysiological study was performed in 638 individuals and had a 41% positivity, but was not a risk predictor on multivariate analysis, leaving only personal history and electrocardiographic pattern correlated with events.

Two years later, a prospective multicenter study,^15^ assessing specifically the accuracy of arrhythmia induced by stimulation and the identification of new risk predictors, evidenced that induced arrhythmia was not an event predictor in a 36-month follow-up (and only 34% of the patients with induced arrhythmia experienced a new induction when repeating the protocol), in addition to the same findings regarding type I ECG and personal history of syncope, and the additional positive finding for ventricular refractory period shorter than 200 ms and QRS fragmentation. Of the 14 events, only 1 showed no spontaneous type I pattern, with a number needed to treat (NNT) of 25.2.

In 2016, however, a systematic review of eight prospective observational studies involving 1312 patients (n ranging from 575 to 23) with BrS, no previous history of sudden death, undergoing ventricular stimulation, showed that induced arrhythmia correlated with events in a mean 38.3-month follow-up, with higher risks for patients induced with one or two extra stimuli.^18^ The overall analysis of data indicates that the electrophysiological study is useful, mainly in patients at intermediate risk, to whom the clinical characteristics cannot provide a dichotomous classification of high or low risk.

In 2017, Sieira et al.^19^ proposed a model of risk classification based on a cohort of 400 patients from a single Belgian center, with mean age and percentage of asymptomatic individuals similar to those of our cohort, in which the clinical factors associated with outcomes were categorized into a score model including the following variables: type I electrocardiographic pattern, history of sudden death of a first-degree relative younger than 35 years, arrhythmia induced on electrophysiological study, syncope, sinus node disease and history of sudden death. In the model proposed, a score equal to or greater than 2 represents high risk for outcome, with positive predictive value of 90%, maintained at 81% when having external validity.

In the present study, the rate of the implantable device events was lower than that reported in the literature, including national studies with patients with BrS,^20^ and the one patient with appropriate therapy received it in the first year of follow-up. Nevertheless, the mean 5-year follow-up showed a temporal gain as compared to many similar studies, allowing for the analysis of events in a larger time window - knowing that the risks are continuous throughout life - with the potential advantage of overcoming occasional inaccurate clinical data, manly family history, because the information is patient-dependent and previous data might not be well characterized in the generation immediately before the proband.

Although controversial, the use of electrophysiological study for stratification has shown to be a useful tool to identify high-risk patients, representing a clear signal that the ventricle is more excitable, and, thus, prone to arrhythmic events.^21^

### Limitations

This study has limitations such as the fact that the cohort is not constituted by patients identified by ECG, but by those, who, according to their attending doctors would benefit from an electrophysiological study for risk stratification, a fact that limited the sample size and can be a bias by selecting patients that raise more concern about future events. Another fact is that, of the 35 patients, 5 did not undergo follow-up at the same institution where the electrophysiological study was performed. In such cases, data were limited to information collected via telephone, with checking up on neither the electronic medical records nor the devices. Moreover, we performed no genetic study of the population assessed, because it is not routinely available in the healthcare system in addition to its costs.

## Conclusion

Brugada syndrome is a potentially fatal arrhythmic condition, and reports on it increased substantially in past years. In this cohort, similarly to the world literature, most patients are of the male sex and had spontaneous type I electrocardiographic pattern. Class IA antiarrhythmic drugs of the Vaughan Williams classification have high rates of electrocardiographic conversion when used for diagnostic challenge. The rate of arrhythmic event was 6.25%, and mortality was lower than that in the literature. The electrophysiological study for risk assessment, although controversial, is currently a useful tool for patient’s stratification, mainly when the clinical characteristics are poor and do not allow for estimating accurately the risks of future events.
